# Delayed motherhood and its determinants among women of reproductive age in five Sub-Saharan African Countries: A multilevel analysis of recent demographic and health surveys (2021–2023)

**DOI:** 10.1371/journal.pone.0326190

**Published:** 2025-08-14

**Authors:** Yilkal Abebaw Wassie, Tekletsadik Tekleslassie Alemayehu, Tsehayu Melak Siyoum, Girum Nakie, Getasew Kibralew, Gebresilassie Tadesse, Zemenu Wube Bayleyegn, Berihun Agegn Mengistie, Gashaw Sisay Chanie, Tesfaye Birhanu Abebe, Leul Dejene Abate, Cherugeta Kebede Asfaw, Mequanint Melesse Bicha, Agazhe Aemro, Gebremariam Wulie Geremew

**Affiliations:** 1 Department of Medical Nursing, School of Nursing, College of Medicine and Health Sciences University of Gondar, Gondar, Ethiopia; 2 Department of Social and Administrative Pharmacy, School of Pharmacy, College of Medicine and Health Sciences, University of Gondar, Gondar, Ethiopia; 3 Department of Neonatal Health Nursing, School of Nursing, College of Medicine and Health Sciences, University of Gondar, Gondar Ethiopia; 4 Department of Psychiatry, College of Medicine and Health Sciences, University of Gondar, Gondar, Ethiopia; 5 Department of General Midwifery, School of Midwifery, College of Medicine and Health Sciences, University of Gondar, Gondar, Ethiopia; 6 Department of Gynecology and Obstetrics, School of Medicine, College of Medicine and Health Sciences, University of Gondar, Gondar Ethiopia; 7 School of Medicine, College of Medicine and Health Sciences, Selale University, Fitche, Ethiopia; 8 Department of Clinical Pharmacology, School of Pharmacy, College of Medicine and Health Sciences, University of Gondar, Gondar, Ethiopia; Babol University of Medical Sciences, IRAN, ISLAMIC REPUBLIC OF

## Abstract

**Introduction:**

In recent years, more women have delayed childbearing until their late twenties or early thirties, a time when reproductive potential declines, thereby making conception more challenging with advancing age. While delayed motherhood is widely reported, there is limited research on its underlying causes in Sub-Saharan Africa. Exploring these factors offers valuable insights for researchers, policymakers, and healthcare providers.

**Method:**

This study used the most recent Demographic and Health Surveys (2021–2023) across five Sub-Saharan Africa countries. A weighted sample of 47,439 women of reproductive age were analyzed using Stata 14. The determinants of delayed motherhood were determined using a multilevel mixed-effects logistic regression model. Statistical significance was determined at p-values <0.05, and results were reported using adjusted odds ratio (AOR) with 95% confidence interval.

**Results:**

A total of 47,439 study participants were enrolled in the study. The mean age was 31.9 years (±8.7 SD), with over half (54.54%) aged between 20 and 35 years. The prevalence of delayed motherhood among women of reproductive age in Sub-Saharan Africa was 61.94%. Individual factors such as did not have occupation (AOR = 1.65, 95% CI: 1.57, 1.73), whose husband did not have occupation (AOR = 1.55, 95% CI: 1.51, 1.78), and those with poor wealth status (AOR = 2.62, 95% CI: 1.58, 3.66) were more likely to delay motherhood. Moreover distance to a health facilities (AOR = 1.04, 95% CI: 1.09, 1.39), urban residence (AOR = 2.48, 95% CI: 1.67, 2.09), and high community illiteracy (AOR = 3.24, 95% CI: 1.55, 5.35) were also significant predictors of delayed motherhood.

**Conclusions:**

This study reveals a significant prevalence of delayed motherhood among women of reproductive age in Sub-Saharan Africa. Major contributing factors include unemployment, poverty, urban residence, limited healthcare access, and community illiteracy. Addressing these disparities requires targeted policies and improved access to education and healthcare services.

## Background

Being a mom is basically a given, even though some women prefer to put off or stay away from getting pregnant and having children. Delayed motherhood refers to women having their first child later in life, a period marked by declining fertility and increased challenges in conceiving [1,2]. In every society, becoming a mother is seen as the most important job that woman can do [3,4]. Age-related infertility in women is particularly vulnerable because aging causes a decrease in the number and quality of egg follicles, which can lead to miscarriages and chromosomal abnormalities [5].

Globally, different articles recognized that a 20-years old healthy woman has a 34% probability of becoming pregnant per cycle [6]. By 30 years of age, this probability is halved to 17% and then falls rapidly to 8% by 37 years of age. At 45 years, the chance of a woman achieving a live birth is as low as 0.5% per cycle [7]. About ten years prior to menopause (the average age for menopause is roughly 51 years), when the menstrual cycle becomes irregular and the biological window for childbearing is quickly closing, conception becomes unlikely [8,9]. Generally, in many nations, the average age of the first birth has outshined 30 years, having risen by 2–4 years over the previous two to three decades [10].

According to fertility trends, the average age at which women give birth to their first child has risen in recent decades. Numerous cultural, societal, and economic settings are contributing to the rise in the number of women giving birth later in life [11,12]. Delaying having children is a complex decision that involves a number of factors, including personal preferences, societal influences, medical concerns, and technological breakthroughs [13]. Existing literature has found that the frequency of postponed pregnancy was consistently linked to women’s educational attainment [14,15], labor market participation, personality traits, attitude [16], and personal preferences; knowledge about fertility [17]; physical and psychological readiness [18]; stable relationships with spouses and other significant individuals; and sociocultural and economic factors [19,20].

In both high-income and low-income countries have recognized that women who are delayed in becoming mothers are more likely to occur a significant risk factor for fetal and perinatal issues [21]. These issues can raise the risk of death and morbidity in the newborn and later years of life. It can also increase the likelihood of health issues for both mothers and children, including higher risks of birth complications and developmental disorders [3,22] like stillbirths, preterm labor, abortion, autism, childhood cancer, and down syndrome, which correlate with advanced maternal and paternal age [23]. Furthermore, the labor market and retirement systems will suffer greatly from low pregnancy rates brought on by postponing childbearing.

The increasing age at which women become pregnant is a universal social issue that has gained prominence in recent decades. Furthermore, scientists contend that postponed maternity is a significant contributing element to lower fertility [24,25]. Several studies have indicated a high prevalence of delayed motherhood. Despite this, there is a scarcity of published articles about delayed motherhood among reproductive age women in Sub Saharan Africa. A thorough analysis of the causes of delayed childbearing can provide researchers, politicians, and healthcare practitioners with important insights into the wider ramifications for people, families, and society [26]. Thus, the purpose of this study was to determine the prevalence and determinant factors of delayed motherhood among reproductive age women in five Sub Saharan Africa.

## Methods

### Patient and public involvement statement

Demographic and Health Surveys (DHS) provided the secondary data used in this investigation.

### Study design, study area, and period

A latest DHS survey multilevel mixed effect analysis was performed using data from the five Sub Saharan African countries between 2021 and 2023. Every five years, a community-based cross-sectional research study is conducted by the DHS to generate updated health and health-related indicators.

### Data source, study population and sampling technique

The Demographic Health Survey (DHS) datasets from 2021 to 2023 for the most current East African nations were used for the secondary data analysis. We utilized DHS surveys from in five Sub Saharan, including: Burkina Faso, Ghana, Kenya, Mozambique and Tanzania. In order to determine the magnitude and contributing determinants of delayed to getting pregnant among reproductive age women in Sub Saharan Africa, the data were appended. Different datasets, such as those for men, women, children, births, and households, are included in the survey for each nation. The DHS uses a stratified two-stage cluster design, where the first stage consists of enumeration regions, and the second stage creates a sample of homes from each enumeration area. Delayed to getting pregnant was the outcome variable, which was created by recoding the variable ever used anything or tried to delay getting pregnant (v302a) from the maternal record (IR) data set. The factors associated with delayed to getting pregnant were identified by using a binary logistic regression model; the factors were reported as an adjusted odds ratio (AOR) with a 95% significance level. In the univariate analysis, variables with p-values <0.25 were considered statistically significant, and all variables with p values <0.05 were deliberated to be at 95% confidence intervals. The study had a weighted sample of 49,439 women in total (**Table 1**).

**Table 1:** Determining the Sample Size for Assessing the Prevalence and Determinants of Delayed Motherhood among women of reproductive age in Five Sub-Saharan African Countries.

Country	Year of survey	Weighted sample (n)	Weighted sample (%)
Burkina Faso	2021	12,252	25.83
Ghana	2022	8,677	18.29
Kenya	2022	9,373	19.76
Mozambique	2021	8,087	17.05
Tanzania	2023	69,050	619.08
Total sample size		47,439	100

### Study variables

#### Dependent variables.

In this study, the dependent variable was delayed to getting pregnant (women postponing having their first child until later in life). It was recorded as “Yes = 1” if the mother was used anything or tried to delay or avoid getting pregnant. On the other hand, it was recorded as “No = 0” if the mother ever never used anything or tried to delay or avoid getting pregnant among reproductive age women (15–49 years).

#### Independent variables.

Since DHS data are hierarchical, independent variables from two sources (variables at the individual and community levels) were taken into consideration for this analysis. The individual-level independent variables were socio economic and information related factors of respondent’s: Maternal age (15–19, 20–35, 36–49), Maternal education (No formal education, Primary, Secondary and Higher), Maternal occupation (Not working, Working), Marital status of the mother (Living with partner, Currently married), Wealth index (Poor, Middle, Rich), Sex of household head (Male, Female), Husband education (No formal education, Primary, Secondary and Higher) and Husband occupation (Not working, Working), **Reproductive health related factors;** Contraceptives (Ever use, Ever not use), Age at first sexual intercourse, Age at first marriage, and Media exposure).

**The community-level variables;** were place of residence (Urban, Rural), Community illiteracy (Low, High), Community-level poverty (Low, High), Community media exposure (Low, High) and country (Burkina Faso, Ghana, Kenya, Mozambique and Tanzania).

### Data processing and statistical analysis

We analyzed DHS data using hierarchical logistic regression models to assess individual and community determinants of delayed motherhood. Using STATA version 14, recent DHS datasets were recorded, cleaned, and examined. The variables in the DHS data are arranged into clusters, with the similarity between variables inside a cluster being greater than that of variables outside of it. Two presumptions were violated in order to use a standard logistic regression model: independent data and equal variance across clusters. This implies that the consideration of between-cluster factors requires the use of a complex model. To investigate the parameters related with delayed to getting pregnant, multilevel mixed-effects logistic regression was employed. Four models are utilized in multilevel mixed effect logistic regression: model I (which only includes individual level factors), model II (which only includes community level variables), model III (which includes both individual and community level variables), and the null model (which uses only outcome variables). To assess the variation in to delay to getting pregnant within the cluster, the null model—which lacks independent variables—was employed. Model I evaluated the relationship between the result variable and the individual-level factors, while Model II evaluated the relationship between the community-level and the outcome variable. Individual and community-level factors were fitted simultaneously with the outcome variable (delayed to getting pregnant) in the final model, known as Model III.

### Random effects

Measures of variation or random effects of the outcome variables were evaluated using the proportionate change in variance (PCV), intra-class correlation coefficient (ICC), and median odds ratio (MOR). The ICC was calculated to determine the proportion of variance attributable to differences between clusters.. Regarding clusters as a random variable, the ICC shows that delayed to getting pregnant varies throughout clusters and may be calculated as follows: ICC = VC/(VC + 3.29)×100%. The MOR is the median value of the odds ratio, utilizing clusters as a random variable, between the highest-risk and lowest-risk areas for delayed to getting pregnant when two clusters are randomly selected; MOR = *e* ^0.95√VC^.

Furthermore, PCV illustrates how factors account for variation in the prevalence of delayed to getting pregnant and is calculated as PCV= (Vnull-VC)/Vnull×100%, where Vnull denotes the variance of the null model and VC denotes cluster level variance (12–14). The probability of delayed to getting pregnant was compared to individual and community level independent variables using the fixed effects method. Using the adjusted odds ratio (AOR) and 95% confidence intervals with a p-value of less than 0.05, it was evaluated and its strength was displayed. Given the hierarchical structure of the model, models were compared using deviation = −2 (log likelihood ratio), with the best-fit model being determined by minimizing deviance. By evaluating the variance inflation factors (VIF), with the help of the models’ variables, multi-collinearity was confirmed.

### Ethical approval and consent to participate

Not applicable. This study utilized secondary data obtained from the Demographic and Health Surveys (DHS) program. Prior to accessing the dataset, permission was granted by the DHS Program/ICF International. The DHS datasets are publicly available and anonymized, and the DHS Program obtained ethical clearance from the relevant national ethics committees and the Institutional Review Board (IRB) of ICF International. As this analysis involved no direct contact with human subjects and used de-identified data, no additional informed consent was required. All procedures were conducted in accordance with the ethical principles outlined in the Declaration of Helsinki. Further information on the DHS data and ethical standards is available at http://www.dhsprogram.com

## Results

### Sociodemographic and economic characteristics of women of reproductive age in five Sub-Saharan Africa

Over-all of 47, 439 women with reproductive age were enrolled in the study. The mean age of the study participant was 31.9 years with ± 8.7 standard deviation. More than half (54.54%) of the study respondents were between the age of 20–35 years. Greater than one third (36.13%) of the study participants did not have formal education. Nearly two third (65.14%) of the study respondents were living in urban areas of sub-Saharan Africa countries, whereas little bit more than one third (66.77%) of the study respondents living in sub-Saharan Africa countries where distance to health facilities is not a big problem to get family planning services. About more than half (52.15%) of women living in sub-Saharan African countries have poor community media exposure (**Table 2**).

**Table 2:** Sociodemographic and economic characteristics of women of reproductive age in five Sub-Saharan Africa countries using DHS 2021-2023.

Variables Independent variables	Category	Frequency (n)	Percent (%)
Maternal age	15-19	2,897	6.11
	20-35	25,873	54.54
	36-49	18,669	39.35
Maternal educational level	No formal education	17,138	36.13
	Primary	15,229	32.10
	Secondary & above	15,072	31.77
Husband education level	No formal education	15,122	31.88
	Primary	15,700	33.10
	Secondary & above	16,617	35.03
Marital status of the women	Living with partner	13,369	71.82
	Currently married	34,070	28.18
Maternal occupation	Not working	28,470	60.01
	Working	18,969	39.99
Husband occupation	Not working	6,596	13.90
	Working	85,141	86.10
Sex of household head	Male	39,106	82.43
	Female	8,333	17.57
Wealth index	Poor	18,848	39.73
	Middle	9,528	20.08
	Rich	19,063	40.18
Age at first sexual intercourse	0-14	8,347	17.60
	15-17	23,448	49.43
	18-49	15,644	32.98
Age at first marriage	0-14	4,523	9.53
	15-17	14,238	30.01
	18-49	28,678	60.45
Use of contraceptive	Ever used	18,017	37.98
	Not ever used	29,422	62.02
Distance to health facility	Not a big problem	31,673	66.77
	Big problem	15,766	33.23
**Community level variables**			
Place of residence	Urban	30,900	65.14
	Rural	16,539	34.86
Community media exposure	Low	26,638	56.15
	High	20,801	43.85
Community poverty	Low	24,058	50.71
	High	23,381	49.29
Community level illiteracy	Low	29,241	61.64
	High	18,198	38.36
Country	Burkina Faso	12,252	25.83
	Ghana	8,677	18.29
	Kenya	9,373	19.76
	Mozambique	8,087	17.05
	Tanzania	9,050	19.08

### Prevalence of delayed motherhood among women of reproductive age in five Sub-Saharan Africa countries

The prevalence of delayed to getting pregnant among reproductive age women in five Sub-Saharan Africa countries was found to be 61.94% (95% CI: (61.5, 62.4)). The magnitude of urban and rural delayed to getting pregnant in five sub-Saharan African countries were found to be 37.53% and 24.42%, respectively.

### Random effect ((Measures of variation) and model fitness

A null model was used to identify whether the data supported the decision to assess randomness at the community level. Results from the null model revealed that there were substantial discrepancy in delayed to getting pregnant between communities, with a variance of 0.5163447 and a P value of 0.000. The variance within clusters contributed 86.43% of the variation in delayed to getting pregnant, whereas the variance across clusters was accountable for 13.57% of the variation. In the null model, the odds of a delayed to getting pregnant differed between higher and lower risk clusters by a factor of 1.98 times. The interclass correlation value for Model I indicated that 8.03% of the variation in delayed to getting pregnant accounts for the difference between communities. Then, with the null model, we used community-level variables to generate Model II. Cluster variations were the basis for 5.51% of the differences in delayed to getting pregnant, according to the ICC value from Model II. In the final model (model III), which recognized approximately 77.5% of the variation in the likelihood of delayed to getting pregnant to both individual and community-level variables (**Table 3**), the likelihood of delayed to getting pregnant varied by 1.38 times across low and high delayed to getting pregnant clusters.

**Table 3:** Model comparison and random effect analysis for delayed motherhood among women of reproductive age in five Sub-Saharan Africa countries.

Parameter	Null model	Model I	Model II	Model III
Variance	0.5163447	0.2872093	0.1919569	0.1162228
ICC	13.57%	8.03%	5.51%	3.41%
MOR	1.98	1.66	1.52	1.38
PCV	Reference	44.37%	62.82%	77.5%
Model fitness				
LLR	−30,654.007	−27,990.308	−29,679.893	−27,368.084
Deviance	61,308.0014	55,980.616	59,359.786	54,736.168

**Key:** ICC: Interacluster correlation, LLR: Logliklihood ratio, MOR: median odds ratio, PCV:proportional change in variance.

### Association of individual- and community-level factors with delayed motherhood among women of reproductive age in SSA

Finally, the final fitted model of multivariable multilevel logistic regression; maternal occupation, husband occupation, wealth index, distance to health facility, please of residence, community level of illiteracy were significantly associated with delayed to getting pregnant among reproductive age women. (Table 4)

**Table 4:** Multivariable multilevel logistic regression analysis of individual- and community-level factors associated with delayed motherhood among women of reproductive age in five Sub-Saharan Africa countries using DHS 2021-2023.

Variables	Category	Model I AOR (95% CI)	Model II AOR (95% CI)	Model III AOR (95% CI)
Individual and community level variables				
Maternal age	15-19	0.86 (0.95,1.03)		0.89 (0.96,1.08)
	20-35	1.02 (0.98,1.11)		1.04 (1.00,1.12)
	36-49	1		1
Maternal educational level	No formal education	1		1
	Primary	1.19 (1.09,1.29)		1.02 (0.81,1.04)
	Secondary & above	1.22 (1.08,1.38)		1.12 (0.97,1.18)
Husband education level	No formal education	1		1
	Primary	1.28 (1.11,1.26)		1.01 (0.92,1.30)
	Secondary & above	1.47 (1.27,1.47)		1.26 (0.45,1.36)
Maternal occupation	Not working	1.72 (1.59,1.74)		**1.65 (1.57,1.73)**
	Working	1		1
Husband occupation	Not working	1.52 (1.49,1.55)		**1.55 (1.51,1.78)**
	Working	1		1
Sex of household head	Male	1		1
	Female	0.84 (0.79,0.88)		0.83 (0.79,0.88)
Wealth index	Poor	2.65 (1.62,3.69)		**2.62 (1.58,3.66)**
	Middle	1.86 (1.81,0.92)		**1.85 (1.79,2.90)**
	Rich	1		1
Age at first sexual intercourse	0-14	1.05 (0.98,1.13)		1.01 (1.00,1.27)
	15-17	1.22 (1.17,1.29)		1.30 (0.23,1.37)
	18-49	1		1
Age at first marriage	0-14	1.04 (0.96,1.13)		1.02 (0.94,1.11)
	15-17	1.02 (0.97,1.08)		1.04 (0.98,1.10)
	18-49	1		
Distance to health facility	Not a big problem	1.06 (1.01,1.11)		**1.04 (1.09,1.39)**
	Big problem	1		1
Community level variables				
Place of residence	Urban		2.62 (1.59,2.05)	**2.48 (1.67,2.09)**
	Rural		1	1
Community media exposure	Low		1	1
	High		1.07 (0.81,1.94)	1.05 (0.85,1.58)
Community level poverty	Low		1	1
	High		1.12 (0.75,1.19)	1.15 (0.78,1.21)
Community level illiteracy	Low		1	1
	High		3.80 (1.63,5.98)	**3.24 (1.55,5.35)**
Country	Burkina Faso		1	1
	Ghana		1.22 (0.14,1.29)	0.78 (0.73,1.85)
	Kenya		1.29 (0.48,1.71)	1.22 (0.56,1.48)
	Mozambique		0.67 (0.64,1.72)	0.53 (0.50,1.58)
	Tanzania		0.97 (0.92,1.03)	0.55 (0.51,1.08)

Women who did not have working were 1.65 times more likely to develop delayed to getting pregnant among reproductive age women as compared to their counterpart (AOR = 1.65, 95% CI: 1.57, 1.73). The odds of delayed to getting pregnant among reproductive age women were 1.55 time more likely to occur women’s whose husband did not have working as compared to their complement (AOR = 1.55, 95% CI: 1.51, 1.78). Delayed to getting pregnant among reproductive age women was 2.62 and 1.85 times more likely to occur among women living in a poor and middle wealth status as compared to women living in a rich wealth index (AOR = 2.62, 95% CI: 1.58,3.66) and (AOR = 1.85, 95% CI: 1.79,2.90) respectively. The odds of delayed to getting pregnant among reproductive age women were 1.04 times more likely to occur among women whose distance to a health facility is not a big problem as compared to women whose distance to a health facility is a big problem (AOR = 1.04, 95% CI: 1.09, 1.39). The odds of delayed to getting pregnant among reproductive age women were 2.48 times more likely to occur among women in urban residences as compared to women living in rural areas (AOR = 2.48, 95% CI: 1.67, 2.09). The odds of delayed to getting pregnant among reproductive age women were 3.24 times more likely to occur women with in a high community illiteracy as compared to women with in a low community illiteracy (AOR = 3.24, 95% CI: 1.55, 5.35).

## Discussion

Delayed motherhood represents an important public health concern, especially in Sub-Saharan Africa. This practice carries both benefits and risks; it may lower the chances of complications linked to teenage pregnancies but can also raise the likelihood of health issues such as gestational diabetes and preeclampsia among older mothers [2]. The aim of this study is to reveal the prevalence and its determinants of delayed motherhood among reproductive age women in five Sub Saharan Africa countries” A recent demographic health survey from 2021 to 2023 multilevel analysis. The overall prevalence of delayed motherhood among reproductive age women in five Sub Saharan Africa countries was found to be 61.94% with (95% CI: (61.5, 62.4)). The prevalence of delayed motherhood found in this study was lower than the findings of a study conducted in India; Vancouver 82% [27], United Kingdom 72% [28], Germany 67% [29]. The possible justification for this discrepancy might be due to cultural norms and societal expectations regarding marriage and family formation can vary significantly between countries [2]. In some cultures, early marriage and childbearing may be more strongly encouraged. It could also may be due to lower levels of education can be associated with earlier marriage and childbearing, as education often delays entry into adulthood and provides opportunities for career advancement [30].

On the other hand, the prevalence of delayed motherhood found in this study was higher than the findings of a study conducted in Colombia 26.14% [31] and Spain 37% [32]. The possible rationalization for this difference may be due to the stage of demographic transition a country is can affect fertility rates. Countries in earlier stages of demographic transition often have higher fertility rates. In addition, there are some individuals in Sub-Saharan Africa who are delaying childbearing due to factors like education and career aspirations and gender inequality can limit women’s ability to make decisions about their reproductive health and future or many women in Sub-Saharan Africa are economically dependent on their husbands or partners, which can limit their autonomy and decision-making power regarding family planning [33].

In the multivariable multilevel mixed effect logistic regression analysis, did not have occupation, whose husband did not have occupation, poor wealth status, distance to a health facility, and community-level variables such as urban residence, high community illiteracy, were significantly associated with delayed to getting pregnant among reproductive age women.

This study examines the associated factors of delayed motherhood among reproductive-age women in five Sub-Saharan African countries. Women who did not have occupation were 1.65 times more likely to develop delayed to getting pregnant among reproductive age women as compared to their counterpart. This result was similar to a study conducted in Japan [34], United Kingdom, Australia [35], and Chile [36]. This might be due to unemployed women may be financially dependent on others, limiting their ability to make independent decisions about family planning. They may have less say in decisions about when to try for a baby, as their financial situation might be controlled by others. Their partner or family might prioritize other financial needs over starting a family. Furthermore it might be due to unemployed women may face social stigma and pressure to marry and have children early to avoid being labeled as leftover women [2,37]. Finally, Even if they desire pregnancy, the lack of independent income can raise significant concerns about covering the costs of prenatal care, childbirth, postnatal care, and raising a child. This can lead to a conscious or unconscious delay.

The current study was found to be that, the odds of delayed to getting pregnant among reproductive age women were 1.55 times more likely to occur women’s whose husband did not have working as compared to their complement. The finding of this study is in line with previous study conducted in Iran [38]. This possibility may be due to; in some cultures, there may be societal pressure on men to be can lead the primary breadwinners, leading to delayed marriage and childbearing. This could be due to unemployment to increased stress and anxiety, which may negatively impact reproductive health [20]. Generally, it likely reflects the complex interplay of financial stress, lifestyle changes, and relationship dynamics that can affect reproductive health [39].

According to this study was found to be that, delayed to getting pregnant among reproductive age women was 2.62 and 1.85 times more likely to occur among women living in a poor and middle wealth status as compared to women living in a rich wealth index. This finding was supported by studies conducted in Ethiopia [40]. This might be due to that women in poor households often face significant financial constraints. This can lead to prioritizing economic survival; the immediate need for food, shelter, and daily expenses may take precedence over family planning considerations. Limited access to nutritious food and healthcare can negatively impact a woman’s reproductive health, potentially making it harder to conceive or leading to a conscious decision to delay pregnancy until health improves [41]. Furthermore, it may be due to financial difficulties are able to strain relationships, potentially leading to delayed marriage or divorce, both of which can impact fertility plans. Moreover, women in lower wealth groups may need to prioritize education and employment to improve their economic situation before starting a family. It could also might be due to the cost of healthcare, including prenatal care and childbirth, can be a significant barrier for women in lower wealth groups [42].

The subject of associated factors, the odds of delayed to getting pregnant among reproductive age women were 1.04 times more likely to occur among women whose distance to a health facility is not a big problem as compared to women whose distance to a health facility is a big problem. This finding was in line with other studies conducted in Netherland [43]. the probably justification for this might be due to;While easy access to healthcare can facilitate family planning and reduce unintended pregnancies, it is not the sole determinant of reproductive behavior. A variety of social, cultural, economic, and personal factors influence women’s decisions about when to have children. Moreover, it might be due to women whose distance to a health facility is not a big problem often have better access to family planning services, allowing women to make informed choices about their reproductive health [44].

The odds of delayed to getting pregnant among reproductive age women were 2.48 times more likely to occur among women in urban residences as compared to women living in rural areas. This finding was coherent with previous studies conducted in Swedish [45]. The possible explanation for this may be due to urban areas often have better access to education, leading to higher levels of education among women. Hence, women in urban areas may have more opportunities for economic independence, allowing them to delay childbearing or urban areas offer more job opportunities, which can delay marriage and childbearing [46]. Furthermore, the higher cost of living in urban areas may make it more difficult for young couples to afford housing and other expenses, leading to delayed childbearing [47].

The odds of delayed to getting pregnant among reproductive age women were 3.24 times more likely to occur women with in a high community illiteracy as compared to women with in a low community illiteracy. This is consistent with the findings from previous studies conducted in Iran [48]. The possible rationalization for this similarity might be due to; the fundamental driver is the information asymmetry created by low literacy. Women in high-illiteracy communities are significantly disadvantaged in accessing and understanding crucial reproductive health information, leading to disempowerment and delayed pregnancy [49]. Additionally, in the absence of reliable information, women are more susceptible to harmful cultural myths and misconceptions about fertility which means women is particularly vulnerable for age related infertility because aging causes a decrease in the number and quality of egg follicles [50]. In conclude, illiteracy can undermine women’s confidence in advocating for their reproductive health needs, leading to passive acceptance of unfavorable circumstances.

## Conclusion

This study highlights a significance prevalence of delayed pregnancy or motherhood among women of reproductive age in five Sub-Saharan Africa countries, with notable variation across communities. The findings underscore that factors such as unemployment, low household wealth, limited access to health facilities, urban residence, and high community illiteracy are significantly associated with delayed motherhood. These determinants reflect broader socioeconomic and structural challenges that influence women’s reproductive choices and opportunities. To address delayed motherhood effectively, policymakers should prioritize strategies that enhance women’s education, economic empowerment, and access to reproductive health services, while also addressing broader structural inequalities across communities.

### Strength and limitation

A strength of the study is that it addresses a significant public health issue, particularly in Sub-Saharan Africa, where delayed motherhood impacts maternal and child health, population dynamics, and socioeconomic development. A limitation of the study is that the findings may not be generalizable to other populations, especially those with different cultural, socioeconomic, and healthcare contexts. Another limitation is that the study includes only women of reproductive age, using secondary DHS Individual Recode (IR) data, which lacks comprehensive information on male partners, thus limiting insights into joint reproductive decision-making. Therefore, future research should incorporate men’s perspectives to provide a more comprehensive understanding of factors influencing delayed motherhood.

## Supporting information

S1 Supplementary file 1. (XLS)

## References

[1] Sulaiman TB, Ibrahim BZ. Legal effects of pregnancy duration on pregnant woman’s flight in air transport: comparative study. Journal of Almaarif University College. 2023;34(2).

[2] Temmesen CG, Faber Frandsen T, Svarre-Nielsen H, Petersen KB, Clemensen J, Andersen HLM. Women’s reflections on timing of motherhood: a meta-synthesis of qualitative evidence. Reprod Health. 2023;20(1):30. doi: 10.1186/s12978-022-01548-x 36755286 PMC9909900

[3] Schmidt L, Sobotka T, Bentzen JG, Nyboe Andersen A, ESHRE Reproduction and Society Task Force. Demographic and medical consequences of the postponement of parenthood. Hum Reprod Update. 2012;18(1):29–43. doi: 10.1093/humupd/dmr040 21989171

[4] Zheng Q, Wang S, Tian X, Liu W, Gao P. Fecal microbiota transplantation confirmed that 919 Syrup reduced the ratio of erucamide to 5-AVAB in hippocampus to alleviate postpartum depression by regulating gut microbes. Front Immunol. 2023;14:1203015. doi: 10.3389/fimmu.2023.1203015 37292211 PMC10244653

[5] Esencan E, Beroukhim G, Seifer DB. Age-related changes in Folliculogenesis and potential modifiers to improve fertility outcomes - A narrative review. Reprod Biol Endocrinol. 2022;20(1):156. doi: 10.1186/s12958-022-01033-x 36397149 PMC9670479

[6] Safdari-Dehcheshmeh F, Noroozi M, Taleghani F, Memar S. Factors Influencing the Delay in Childbearing: A Narrative Review. Iran J Nurs Midwifery Res. 2023;28(1):10–9. doi: 10.4103/ijnmr.ijnmr_65_22 37250942 PMC10215553

[7] Sabdilon A. Transitions of motherhood among first time late mothers: A qualitative investigation. Asian Journal of Multidisciplinary Studies. 2018;1(1).

[8] Hull MG, Fleming CF, Hughes AO, McDermott A. The age-related decline in female fecundity: a quantitative controlled study of implanting capacity and survival of individual embryos after in vitro fertilization. Fertil Steril. 1996;65(4):783–90. doi: 10.1016/s0015-0282(16)58214-4 8654639

[9] Joseph KS, Allen AC, Dodds L, Turner LA, Scott H, Liston R. The perinatal effects of delayed childbearing. Obstet Gynecol. 2005;105(6):1410–8. doi: 10.1097/01.AOG.0000163256.83313.36 15932837

[10] Ibarra-Nava I, Choudhry V, Agardh A. Desire to delay the first childbirth among young, married women in India: A cross-sectional study based on national survey data. BMC Public Health. 2020;20(1):350. 32183765 10.1186/s12889-020-8402-9 PMC7079505

[11] Šprocha B, Tišliar P, Šídlo L. A cohort perspective on the fertility postponement transition and low fertility in Central Europe. Moravian Geographical Reports. 2018;26(2):109–20. doi: 10.2478/mgr-2018-0009

[12] Benzies K, Tough S, Tofflemire K, Frick C, Faber A, Newburn-Cook C. Factors influencing women’s decisions about timing of motherhood. J Obstet Gynecol Neonatal Nurs. 2006;35(5):625–33. doi: 10.1111/j.1552-6909.2006.00079.x 16958718

[13] Majcher M, et al. Mental health issues during pregnancy-overview of the current knowledge. Journal of Education, Health and Sport. 2023;29(1):52–8.

[14] Williamson LEA, Lawson KL, Downe PJ, Pierson RA. Informed reproductive decision-making: the impact of providing fertility information on fertility knowledge and intentions to delay childbearing. J Obstet Gynaecol Can. 2014;36(5):400–5. doi: 10.1016/S1701-2163(15)30585-5 24927291

[15] Neels K, Murphy M, Ní Bhrolcháin M, Beaujouan É. Rising Educational Participation and the Trend to Later Childbearing. Popul Dev Rev. 2017;43(4):667–93. doi: 10.1111/padr.12112 29398739 PMC5767733

[16] Billari FC, Philipov D, Testa MR. Attitudes, norms and perceived behavioural control: Explaining fertility intentions in bulgaria/attitudes, normes et contrôle perçu du comportement: Une explication des intentions de fécondité en bulgarie. European Journal of Population/Revue Europénne de Démographie. 2009:439–65.

[17] Tough S, Tofflemire K, Benzies K, Fraser-Lee N, Newburn-Cook C. Factors influencing childbearing decisions and knowledge of perinatal risks among Canadian men and women. Matern Child Health J. 2007;11(2):189–98. doi: 10.1007/s10995-006-0156-1 17237994

[18] Kearney AL, White KM. Examining the psychosocial determinants of women’s decisions to delay childbearing. Hum Reprod. 2016;31(8):1776–87. doi: 10.1093/humrep/dew124 27240695

[19] Sørensen NO, et al. Fertility awareness and attitudes towards parenthood among danish university college students. Reproductive Health. 2016;13(1):1–10. 27964723 10.1186/s12978-016-0258-1 PMC5154162

[20] Zabak S, Varma A, Bansod S, Pohane MR. Exploring the Complex Landscape of Delayed Childbearing: Factors, History, and Long-Term Implications. Cureus. 2023;15(9):e46291. doi: 10.7759/cureus.46291 37915872 PMC10616531

[21] Lawson G, Fletcher R. Delayed fatherhood. J Fam Plann Reprod Health Care. 2014;40(4):283–8. doi: 10.1136/jfprhc-2013-100866 24958072

[22] Silva PYF, Lima da Cruz MC, Guerra Azevedo I, Moreira RS, Sousa KG, Pereira SA. Risk of Global Developmental Delay in Infants Born from Mothers with COVID-19: A Cross-Sectional Study. Int J Womens Health. 2023;15:467–74. doi: 10.2147/IJWH.S389291 37033123 PMC10075265

[23] Myrskylä M, Barclay K, Goisis A. Advantages of later motherhood. Der Gynakologe. 2017;50(10):767. 29070913 10.1007/s00129-017-4124-1 PMC5633623

[24] Qin X, Zhang W, Xu S, Ma M, Fan X, Nie X, et al. Prevalence and risk factors of anxious and depressive symptoms in first-trimester females and their partners: a study during the pandemic era of COVID-19 in China. BMC Psychiatry. 2023;23(1):134. doi: 10.1186/s12888-023-04621-2 36869299 PMC9982791

[25] Petersen KB. Individual fertility assessment and counselling in women of reproductive age. Dan Med J. 2016;63(10):B5292. 27697140

[26] Behboudi-Gandevani S, Ziaei S, Farahani FK, Jasper M. The Perspectives of Iranian Women on Delayed Childbearing: A Qualitative Study. J Nurs Res. 2015;23(4):313–21. doi: 10.1097/JNR.0000000000000084 26562463

[27] Wiebe E, Chalmers A, Yager H. Delayed motherhood: understanding the experiences of women older than age 33 who are having abortions but plan to become mothers later. Can Fam Physician. 2012;58(10):e588-95. 23064938 PMC3470537

[28] Proudfoot S, Wellings K, Glasier A. Analysis why nulliparous women over age 33 wish to use contraception. Contraception. 2009;79(2):98–104. 19135565 10.1016/j.contraception.2008.09.005

[29] Simpson R. Delayed childbearing and childlessness. Fertility, Living Arrangements, Care and Mobility: Understanding Population Trends and Processes - Volume 1. 2009. p. 23–40.

[30] Omoeva C, Hatch R. Teenaged, married, and out of school: Effects of early marriage and childbirth on school exit in Eastern Africa. Prospects. 2020;52(3–4):299–324. doi: 10.1007/s11125-020-09517-7

[31] Molina-García L, Hidalgo-Ruiz M, Cocera-Ruíz EM, Conde-Puertas E, Delgado-Rodríguez M, Martínez-Galiano JM. The delay of motherhood: Reasons, determinants, time used to achieve pregnancy, and maternal anxiety level. PLoS One. 2019;14(12):e0227063. doi: 10.1371/journal.pone.0227063 31887126 PMC6936780

[32] Ballesteros-Meseguer C, et al. Adecuación entre la práctica clínica obstétrica en el hospital clínico universitario virgen de la arrixaca (murcia) y las recomendaciones de la estrategia de atención al parto normal. Matronas Profesión. 2015;16(4).

[33] Darteh EKM, Dickson KS, Doku DT. Women’s reproductive health decision-making: A multi-country analysis of demographic and health surveys in sub-Saharan Africa. PLoS One. 2019;14(1):e0209985. doi: 10.1371/journal.pone.0209985 30625212 PMC6326492

[34] Molina-García L, Hidalgo-Ruiz M, Cocera-Ruíz EM, Conde-Puertas E, Delgado-Rodríguez M, Martínez-Galiano JM. The delay of motherhood: Reasons, determinants, time used to achieve pregnancy, and maternal anxiety level. PLoS One. 2019;14(12):e0227063. doi: 10.1371/journal.pone.0227063 31887126 PMC6936780

[35] Guardiola M. La maternidad tardía: expresión contemporánea del patriarcado occidental. Antropología Experimental. 2016.

[36] Gindoff PR, Jewelewicz R. Reproductive potential in the older woman. Fertil Steril. 1986;46(6):989–1001. doi: 10.1016/s0015-0282(16)49869-9 3536609

[37] Friedline T, Chen Z, Morrow S. Families’ Financial Stress & Well-Being: The Importance of the Economy and Economic Environments. J Fam Econ Issues. 2021;42(Suppl 1):34–51. doi: 10.1007/s10834-020-09694-9 32837140 PMC7362317

[38] Javadifar N, Majlesi F, Nikbakht A, Nedjat S, Montazeri A. Journey to Motherhood in the First Year After Child Birth. J Family Reprod Health. 2016;10(3):146–53. 28101116 PMC5241359

[39] Sharma R, Biedenharn KR, Fedor JM, Agarwal A. Lifestyle factors and reproductive health: taking control of your fertility. Reprod Biol Endocrinol. 2013;11:66. doi: 10.1186/1477-7827-11-66 23870423 PMC3717046

[40] Gurara MK, Draulans V, Van Geertruyden J-P, Jacquemyn Y. Determinants of maternal healthcare utilisation among pregnant women in Southern Ethiopia: a multi-level analysis. BMC Pregnancy Childbirth. 2023;23(1):96. doi: 10.1186/s12884-023-05414-x 36739369 PMC9898958

[41] Odoms-Young A, Brown AGM, Agurs-Collins T, Glanz K. Food Insecurity, Neighborhood Food Environment, and Health Disparities: State of the Science, Research Gaps and Opportunities. Am J Clin Nutr. 2024;119(3):850–61. doi: 10.1016/j.ajcnut.2023.12.019 38160801 PMC10972712

[42] Hussen NM, Workie DL. Multilevel analysis of women’s education in Ethiopia. BMC Womens Health. 2023;23(1):197. doi: 10.1186/s12905-023-02380-6 37106332 PMC10142427

[43] Mills M, Rindfuss RR, McDonald P, te Velde E, ESHRE Reproduction and Society Task Force. Why do people postpone parenthood? Reasons and social policy incentives. Hum Reprod Update. 2011;17(6):848–60. doi: 10.1093/humupd/dmr026 21652599 PMC3529638

[44] Dotse-Gborgbortsi W, Nilsen K, Ofosu A, Matthews Z, Tejedor-Garavito N, Wright J, et al. Distance is “a big problem”: a geographic analysis of reported and modelled proximity to maternal health services in Ghana. BMC Pregnancy Childbirth. 2022;22(1):672. doi: 10.1186/s12884-022-04998-0 36045351 PMC9429654

[45] Schytt E, Nilsen ABV, Bernhardt E. Still childless at the age of 28 to 40 years: a cross-sectional study of Swedish women’s and men’s reproductive intentions. Sex Reprod Healthc. 2014;5(1):23–9. doi: 10.1016/j.srhc.2013.11.001 24472386

[46] Dhamija G, Roychowdhury P, Shankar B. Does urbanization empower women? Evidence from India. J Popul Econ. 2025;38(1). doi: 10.1007/s00148-025-01085-4

[47] Leishman C, Liang W, Sim N. The impact of urban population on housing cost: The case of australia. npj Urban Sustain. 2023;3(1):57.

[48] Safdari-Dehcheshmeh F, et al. Factors influencing the delay in childbearing: A narrative review. Iran J Nurs Midwifery Res. 2023;28(1):10–9. 37250942 10.4103/ijnmr.ijnmr_65_22 PMC10215553

[49] Matovelo D, Ndaki P, Yohani V, Laisser R, Bakalemwa R, Ndaboine E, et al. Why don’t illiterate women in rural, Northern Tanzania, access maternal healthcare?. BMC Pregnancy Childbirth. 2021;21(1):452. doi: 10.1186/s12884-021-03906-2 34182949 PMC8240192

[50] John JN, Gorman S, Scales D, Gorman J. Online Misleading Information About Women’s Reproductive Health: A Narrative Review. J Gen Intern Med. 2025;40(5):1123–31. doi: 10.1007/s11606-024-09118-6 39511120 PMC11968640

